# Effect of a Mediterranean type diet on inflammatory and cartilage degradation biomarkers in patients with osteoarthritis

**DOI:** 10.1007/s12603-016-0806-y

**Published:** 2016-09-28

**Authors:** J. Dyer, G. Davison, S. M. Marcora, Alexis R. Mauger

**Affiliations:** School of Sport and Exercise Sciences, The Medway Campus, University of Kent, Kent, ME4 4AG UK

**Keywords:** Diet, sCOMP, AIMS2, IL-1α, osteoarthritis (OA), Mediterranean diet

## Abstract

**Objectives:**

To investigate the effects of a Mediterranean type diet on patients with osteoarthritis (OA).

**Participants:**

Ninety-nine volunteers with OA (aged 31 - 90 years) completed the study (83% female).

**Setting:**

Southeast of England, UK.

**Design:**

Participants were randomly allocated to the dietary intervention (DIET, n = 50) or control (CON, n = 49). The DIET group were asked to follow a Mediterranean type diet for 16 weeks whereas the CON group were asked to follow their normal diet.

**Measurements:**

All participants completed an Arthritis Impact Measurement Scale (AIMS2) pre-, mid- and post- study period. A subset of participants attended a clinic at the start and end of the study for assessment of joint range of motion, ROM (DIET = 33, CON = 28), and to provide blood samples (DIET = 29, CON = 25) for biomarker analysis (including serum cartilage oligomeric matrix protein (sCOMP) (a marker of cartilage degradation) and a panel of other relevant biomarkers including pro- and anti-inflammatory cytokines).

**Results:**

There were no differences between groups in the response of any AIMS2 components and most biomarkers (p > 0.05), except the pro-inflammatory cytokine IL-1α, which decreased in the DIET group (~47%, p = 0.010). sCOMP decreased in the DIET group by 1 U/L (~8%, p = 0.014). There was a significant improvement in knee flexion and hip rotation ROM in the DIET group (p < 0.05).

**Conclusions:**

The average reduction in sCOMP in the DIET group (1 U/L) represents a meaningful change, but the longer term effects require further study.

## Introduction

Often the first line of treatment for someone presenting with OA is a combination of pharmacological interventions, including non-steroidal anti-inflammatory drugs (NSAIDs) and analgesics (1). This can provide initial short-term benefit through an improvement in pain management and a delay to surgical intervention (2). However, this does not prevent OA progression and, as it progresses, reliance on NSAIDs to control pain presents additional complications. Indeed, the long-term use of pharmacological agents (particularly NSAIDs) is associated with numerous side effects (3). Consequently, such treatments are at most, moderately effective at reducing discomfort in the early stages of the disease (1). Therefore, there is a need for safe and effective alternative treatments that not only provide symptom relief but also slow the development of the disease.

It is now recognised that nutrition can play a beneficial role in some chronic diseases (4). Eating a diet high in Trans and saturated fats can increase OA risk factors, as well as exacerbate current osteoarthritic symptoms (5). Given the potential impact of diet on OA, empirical research is required to validate whether the compliance with basic dietary advice alone may improve OA symptoms and/or relevant biomarkers. A Mediterranean type diet (e.g. abundant in vegetables, fruits, beans, whole grains, olive oil and fish, and less red meat than typical Western diets) (6) has been linked with reductions in joint inflammation in patients with rheumatoid arthritis (7), so further work is warranted to examine this type of diet in patients with OA. Hence, the aim of the current study was to investigate the effects of a short-term (16 week) dietary intervention (in accordance with a Mediterranean type diet) on perceptual, functional and serum biomarkers in subjects with OA.

## Methods

### Participants

A total of 124 volunteers with a clinician diagnosis of Osteoarthritis (aged 31-90 years) were recruited with 99 completing (83% female) the study.

Participants were excluded if they had another health condition or co-morbidity that would make them unable to follow the dietary intervention; if participating in any other intervention based research; or if they had prior knowledge or involvement with the organisation providing the dietary advice (Arthritis Action, UK) to reduce the potential for control group contamination/loss of blinding.

### Design

Participants were randomly allocated to one of two groups: the dietary intervention (DIET) group (n = 50) or the control (CON) group (n = 49). From the 99 participants, a subset of 61 participants (DIET = 33, CON = 28) attended a research clinic located at one of 3 locations in the southeast of England, UK (Medway, London, Eastbourne) at the start and at the end of the study. This subset was tested for their range of motion at the knee and hip joints and mobility of the index finger. Of these 61, blood samples were obtained from 54 participants (DIET = 29, CON = 25) for the assessment of biomarkers (samples could not be obtained from 7), and (due to equipment failure) body mass was measured for 39 of these participants (DIET = 22, CON = 17).

### DIET group

Participants mean age was 66 ± 11 y (<40 y n=2; 41-60 y n=13; 61-80 y n=29; >80 n=5) which comprised of n=38 females and n=11 males. Nutritional information and dietary advice were provided consistent with a Mediterranean diet. Support was also offered from a registered Dietician (employed by Arthritis Action), via telephone, which included answering questions and providing guidance on the diet. This intervention/advice is consistent with being member of Arthritis Action and considered advisable when implementing the Mediterranean diet to people from non-Mediterranean countries (8).

### CON group

Participants mean age was 60 ± 12 y (<40 y n=2; 41-60 y n=22; 61-80 y n=22; >80 n=4) which comprised of n=44 females and n=6 males. Participants in this group followed no intervention and were not aware of Arthritis Action.

### Food diary and symptom questionnaires

All participants were asked to complete a 7-day food diary (as a food frequency questionnaire) and an Arthritis Impact Measurement Scale (AIMS2) questionnaire at the start (pre-intervention), mid-point (2 months) and end (4 months) of the study. All questionnaires were mailed to participants with Freepost return. Interpretation of the food diaries and AIMS2 questionnaires was completed by an Arthritis Action staff member who was blinded to the group allocation. A compliance score (from 0-100) was calculated for each food diary.

### Analytical methods

Venous blood samples were obtained by venepuncture (with minimal stasis) into untreated Vacutainer tubes (Becton Dickinson, Oxford, UK). After clotting, samples were centrifuged (1500 × g, 4°C) to obtain serum. All samples were processed within 8 hours. Serum samples were aliquotted and stored at - 80°C for later analysis of selected biomarkers. All samples were thawed only once prior to analysis. The primary biomarker was serum cartilage oligomeric matrix protein (sCOMP), because of previously reported relationships with OA and early-stage OA (9). sCOMP was determined on one serum aliquot using an enzyme-linked immunosorbent assay (ELISA kit, AN-14-1006-71, AnaMar AB, Goteborg, Sweden). Secondary biomarkers included inflammatory cytokines, chemokines and growth factors because of established relationships with OA (10, 11). These were performed using multiplex panels, on a second serum aliquot, which also included some other markers (high-sensitivity panel with IL-1β, IL-1α, IL-2, IL-4, IL-6, IL-8, IL-10, IFN-γ, TNF-α, VEGF and EGF; and panel with soluble receptors IL-6sR, IL-2sR, TNF-sR1 and TNF-sR2, plus MCP-1, and MMP-9) using an Evidence Investigator System and biochip (EV3661 and EV3623, Randox, County Antrim, UK).

### Ethical Approval

This study was conducted according to the Declaration of Helsinki (2008, including 2013 amendments) and all procedures were approved by SSES Research Ethics Advisory Group, University of Kent (Reference Number: Prop 56_2012_2013). Written informed consent was obtained from all participants.

### Data Analysis

All data are presented as Means ± SD. Data were checked for standard assumptions for each statistical test prior to analysis. Where these were violated, data was either log or square root transformed prior to analysis. Differences between groups for changes over time (e.g. pre-, mid-, post-intervention) were assessed using mixed ANOVA (time×group). If data could not be normalised, non-parametric tests were used. All analysis was completed using SPSS (IBM SPSS Statistics for Windows, Version 21.0, Armonk, NY: IBM Corp.), with significance accepted when p < 0.05.

## Results

There was a significant difference between groups in the proportion of subjects changing dietary behaviour from low to high compliance (χ2 p < 0.001): 30 out of the 50 participants in the DIET group, and only 8 of the 49 participants in the CON group, improved their compliance score from below to above 65 (defined as the threshold for high compliance in this study). This improvement in compliance in the diet group, with no change in control group was also evident for each of the subset analyses described below (body mass; χ2 p = 0.012; biomarkers; χ2 p = 0.014; range of motion; χ2 p = 0.008). There was a significant group × time interaction (p = 0.008) for body mass with post hoc analysis revealing a significant reduction in the DIET group (70.4 ± 13.1 to 68.9 ± 12.6 kg, p = 0.012), but no change in the CON group (71.6 ± 17.4 to 72.4 ± 16.6 kg, p = 0.210).

### Biomarkers

There were no differences between groups in the response of any of the biomarkers measured, except the pro-inflammatory cytokine IL-1α (interaction p = 0.019), which decreased pre-to post-intervention in the DIET group compared to no change in CON (Table 1). For sCOMP a strong trend was evident (interaction p = 0.057) and if explored further, at the group level, a significant pre-to post-intervention decrease was evident in the DIET group with no change in the CON group (Table 1).

**Table 1:**
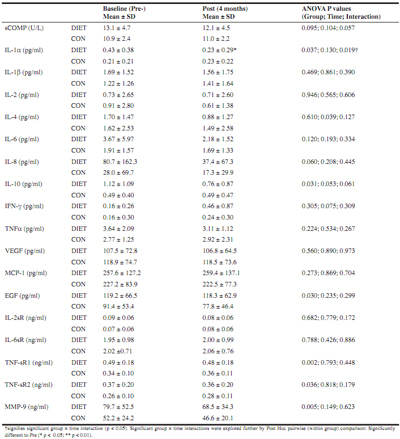
Serum biomarkers

**Table 2:**
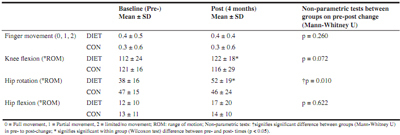
Range of motion for most affected limb

**Table 3:**
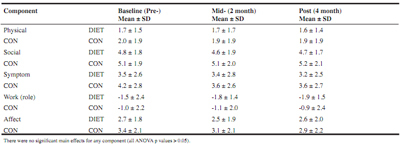
AIMS2 ratings

### AIMS2

No significant interactions were found in any of the AIMS2 components across the study period between groups (all ANOVA p > 0.05, see Table 3).

### Range of motion

ROM- could not be normalised so non-parametric tests were used as detailed in Table 2.

## Discussion

The main findings of the present study were that the dietary intervention was successful at changing eating behaviours in the DIET group, and this was associated with weight loss. The inflammatory cytokine IL-1α (which has been implicated in the pathogenesis of OA, 10) was significantly reduced in the DIET group (but not CON). However, IL-1α concentration was higher pre-intervention in the DIET group (compared to CON). A similar, albeit non-significant (interaction P = 0.057) pattern was evident for sCOMP. When investigating groups separately, a decrease in sCOMP was evident only in the DIET group. This biomarker is widely regarded as a marker of cartilage degradation but has also recently been suggested to indicate severity of synovitis (9). However, as with IL-1α, we also observed a higher concentration of sCOMP at the pre-intervention time in the DIET group, compared to CON. Nevertheless, a decrease for both of these markers in the DIET group can be interpreted as beneficial, although further study is required to establish whether these decreases would continue further with a more prolonged intervention and thus provide long-term benefit. It is worthy of note that sCOMP has been identified as useful early-stage OA marker (9), so the reductions seen in the DIET group in a relatively short intervention period may be of more long-term, clinical, benefit. Changes in this marker may precede subsequent changes in physiological, functional and/or perceptual markers, which explain the lack of improvement in the self-reported AIMS2 questionnaire scores in the present study. Indeed, relatively small changes in biomarkers have been shown to precede any radiographic evidence of joint destruction (13). As such, benefits to perceptual markers (e.g. AIMS2) may manifest after a more prolonged period. Significant improvements were seen in the DIET group for ROM at the hip (rotation) and knee (flexion), which may represent a functional benefit of the dietary intervention which has practical relevance. Taken together, these results suggest potential further benefit if the dietary improvements were continued in the longer-term, although this requires further study. Indeed, a longitudinal study (6 year follow-up) by Kumm et al. (14) demonstrated that sCOMP concentrations were predictive of subsequent progressive knee osteophytosis over the first 3 years of the study period. In this study Kumm et al. (14) demonstrated that an increase of 1 U/L in sCOMP concentration was associated with a 33% higher risk of knee osteophyte progression. Hence, the average reduction in the DIET group (1 U/L) represents a meaningful difference in this marker, which could be of long term clinical benefit.

Body mass reductions have been shown to improve pain and function in those suffering from OA (15, 16) and have also been shown to parallel reductions in cartilage degradation biomarkers such as sCOMP (16). However, Riddle and Stratford (15) suggested that a body mass reduction in excess of 10% is required. It is noteworthy, therefore, that the reduction in sCOMP observed in the DIET group (~8%) was comparable to the 10% reduction reported by Bartels et al (16) despite the relatively small parallel reduction in body mass (~2.2%) observed in the current study. Whilst reductions in body mass alone are likely significant contributors, the present findings suggest that other factors in the DIET group also contribute to the reduction in sCOMP. Nevertheless, body mass reduction is clearly important and this study has demonstrated that adherence to a Mediterranean type diet may have practical and clinical benefit for individuals with OA. Hence, a longer intervention period, and the addition of interventions that promote greater weight loss (e.g. physical activity) may be further beneficial, and so further study is warranted to determine the effectiveness of such interventions.

In conclusion, the dietary intervention was successful at changing eating behaviours and this was associated with weight loss. In addition, the average reduction in sCOMP in the DIET group (1 U/L) represents a meaningful change in relation to OA (9, 14), but the longer term effects of this intervention require further study.

*Funding:* This study was funded by Arthritis Action (grant number 35200)

*Acknowledgments:* This study was funded by a research grant from Arthritis Action (formerly the Arthritic Association), UK.

*Compliance with Ethical Standards:* This study was conducted according to the guidelines laid down in the Declaration of Helsinki (2008, including 2013 amendments) and all procedures were approved by the University Research Ethics Committee. Written informed consent was obtained from all participants. No animals were used in this study.

*Conflicts of interest:* Drs. Dyer, Davison, Marcora and Mauger report grants from Arthritis Action, during the conduct of the study. There are no other conflicts of interest.

## References

[1] McCarberg B., Tenzer P. (2013). Complexities in the pharmacologic management of osteoarthritis pain. Curr Med Res Opin.

[2] Creamer P. (2000). Osteoarthritis pain and its treatment. Curr Opin Rheumatol..

[3] Klinge S.A., Sawyer G.A. (2013). Effectiveness and safety of topical versus oral nonsteroidal anti-inflammatory drugs: a comprehensive review. Phys Sportsmed.

[4] Lopez H.L. (2012). Nutritional interventions to prevent and treat osteoarthritis. Part I: focus on fatty acids and macronutrients. PM R..

[5] Jungmann P.M., Kraus M.S., Alizai H. (2013). Association of metabolic risk factors with cartilage degradation assessed by T2 relaxation time at the knee: data from the osteoarthritis initiative. Arthritis Care Res.

[6] Bach-Faig A., Berry E.M., Lairon D., Reguant J., Trichopoulou A., Dernini S., Medina F.X., Battino M., Belahsen R., Miranda G., Serra-Majem L. (2011). Mediterranean diet pyramid today. Science and cultural updates. Public Health Nutr.

[7] Sköldstam L., Hagfors L., Johansson G. (2003). An experimental study of a Mediterranean diet intervention for patients with rheumatoid arthritis. Ann Rheum Dis.

[8] Middleton G., Keegan R., Smith M.F., Alkhatib A., Klonizakis M. (2015). Brief Report: Implementing a Mediterranean Diet Intervention into a RCT: Lessons Learned from a Non-Mediterranean Based Country. J Nutr Health Aging.

[9] Van Spil W.E., Jansen N.W.D., Bijlsma J.W.J. (2012). Clusters within a wide spectrum of biochemical markers for osteoarthritis: data from CHECK, a large cohort of individuals with very early symptomatic osteoarthritis. Osteoarthritis Cartilage.

[10] Sokolove J., Lepus C.M. (2013). Role of inflammation in the pathogenesis of osteoarthritis: latest findings and interpretations. Ther Adv Musculoskelet Dis.

[11] Penninx B.W.J.H., Abbas H., Ambrosius W. (2004). Inflammatory markers and physical function among older adults with knee osteoarthritis. J Rheumatol.

[12] Towle C.A., Hung H.H., Bonassar L.J., Treadwell B. V., Mangham D.C. (1997). Detection of interleukin-1 in the cartilage of patients with osteoarthritis: a possible autocrine/paracrine role in pathogenesis. Osteoarthritis Cartilage.

[13] Sharif M., Kirwan J., Charni N., Sandell L.J., Whittles C., Garnero P. (2007). A 5-yr longitudinal study of type IIA collagen synthesis and total type II collagen degradation in patients with knee osteoarthritis—association with disease progression. Rheumatology.

[14] Kumm J., Tamm A., Lintrop M., Tamm A. (2013). The value of cartilage biomarkers in progressive knee osteoarthritis: cross-sectional and 6-year follow-up study in middleaged subjects. Rheumatol Int.

[15] Riddle D.L., Stratford P.W. (2013). Body weight changes and corresponding changes in pain and function in persons with symptomatic knee osteoarthritis: a cohort study. Arthritis Care Res.

[16] Bartels E.M., Christensen R., Christensen P. (2014). Effect of a 16 weeks weight loss program on osteoarthritis biomarkers in obese patients with knee osteoarthritis: a prospective cohort study. Osteoarthritis Cartilage..

